# Immunocytochemically detected free peritoneal tumour cells (FPTC) are a strong prognostic factor in gastric carcinoma

**DOI:** 10.1038/sj.bjc.6690096

**Published:** 1999-02

**Authors:** H Nekarda, C Geß, M Stark, J D Mueller, U Fink, U Schenck, J R Siewert

**Affiliations:** Department of Surgery, Klinikum rechts der Isar, Technical University of Munich, Munich, D-81675, Germany; Institute for Statistics and Epidemiology, Klinikum rechts der Isar, Technical University of Munich, Munich, D-81675, Germany; Division of Cytology, Institute of Pathology, Klinikum rechts der Isar, Technical University of Munich, Munich, D-81675, Germany

**Keywords:** gastric cancer, prognosis, isolated tumour cells, staging lavage, immunocytology

## Abstract

We prospectively investigated the prognostic significance of free peritoneal tumour cells (FPTC) in a series of 118 patients with completely resected gastric carcinoma. Immunocytochemistry with the monoclonal antibody Ber-Ep4 was performed on cytospins from intraoperative peritoneal lavage specimens. Twenty-three patients (20%) had FPTC which was significantly correlated with pT and pN categories, stage, tumour size, lymphatic invasion, Laurèn and WHO classifications and perigastric adipose tissue metastases. The median survival time for all FPTC positive compared with negative patients was significantly shorter (11 compared with > 72 months), with estimated 5-year survival rates of 8% vs. 60%. None of the patients with FPTC had an early gastric cancer. In advanced tumour subgroups without and with serosal invasion (*n* = 59 and 35), there were 19% and 34% with FPTC. Multivariate survival analysis showed nodal status, FPTC, mesenteric lymphangiosis, and lymph node metastasis to the compartment III to be independent prognostic factors with relative risks of 6.6, 4.5, 2.9 and 2.2 respectively. Recurrent disease occurred in 91% of FPTC-positive and in 38% of FPTC-negative patients. FPTC had a positive predictive value of 91% and a specificity of 97% for tumour recurrence. FPTC is a strong negative, independent prognostic indicator for survival in gastric carcinoma. © 1999 Cancer Research Campaign


British Journal of Cancer (1999) **79**(3/4), 611–619
© 1999 Cancer Research Campaign
Article no. bjoc.1998.0096

### Immunocytochemically detected free peritoneal tumour cells (FPTC) are a strong prognostic factor in gastric carcinoma

**H Nekarda[1], C Geß[1], M Stark[2], JD Mueller[1], U Fink[1], U Schenck[3] and JR Siewert[1]**

[1]Department of Surgery, Klinikum rechts der Isar, Technical University of Munich, D-81675 Munich, Germany; [2]Institute for Statistics and Epidemiology, Klinikum rechts der Isar, Technical University of Munich, D-81675 Munich, Germany; [3]Division of Cytology, Institute of Pathology, Klinikum rechts der Isar, Technical University of Munich, D-81675 Munich, Germany

**Summary** We prospectively investigated the prognostic significance of free peritoneal tumour cells (FPTC) in a series of 118 patients with completely resected gastric carcinoma. Immunocytochemistry with the monoclonal antibody Ber-Ep4 was performed on cytospins from intraoperative peritoneal lavage specimens. Twenty-three patients (20%) had FPTC which was significantly correlated with pT and pN categories, stage, tumour size, lymphatic invasion, Laurèn and WHO classifications and perigastric adipose tissue metastases. The median survival time for all FPTC positive compared with negative patients was significantly shorter (11 compared with > 72 months), with estimated 5-year survival rates of 8% vs. 60%. None of the patients with FPTC had an early gastric cancer. In advanced tumour subgroups without and with serosal invasion (*n* = 59 and 35), there were 19% and 34% with FPTC. Multivariate survival analysis showed nodal status, FPTC, mesenteric lymphangiosis, and lymph node metastasis to the compartment III to be independent prognostic factors with relative risks of 6.6, 4.5, 2.9 and 2.2 respectively. Recurrent disease occurred in 91% of FPTC-positive and in 38% of FPTC-negative patients. FPTC had a positive predictive value of 91% and a specificity of 97% for tumour recurrence. FPTC is a strong negative, independent prognostic indicator for survival in gastric carcinoma.

**Keywords:** gastric cancer; prognosis; isolated tumour cells; staging lavage; immunocytology

Although it has seen a decrease in incidence, gastric carcinoma is still a significant cause of cancer death, with an overall 5-year survival rate of approximately 15%, and 48% for patients with complete (R0-UICC) resection of their tumour (Allum et al, 1989; Bollschweiler et al, 1993; Roder et al, 1993). Even considering epidemiological differences and earlier diagnoses, it still appears that the more radical surgery routinely practiced in Japan may be beneficial for patients with gastric carcinoma (Noguchi et al, 1989; Bollschweiler et al, 1993; Bonenkamp et al, 1993; Thompson et al, 1993), although there is still some controversy regarding the benefit of extended lymphadenectomy (Siewert et al, 1993; Bonenkamp et al, 1995; Roder et al, 1995). One argument in favour of extended lymphadenectomy is that microscopic deposits of tumour can be frequently detected by immunohistochemistry in perigastric lymph nodes, diagnosed by routine histological methods to be free of tumour (Siewert et al, 1996).

Despite these extended procedures, tumour recurrence is not infrequent in patients who have had a complete resection of their gastric carcinoma. One of the most common (30–50%) (Boku et al, 1990) and earliest forms of tumour recurrence is disseminated peritoneal carcinosis, which predicts a very poor prognosis for the patient (Spratt, 1986; Allum et al, 1989; Nakamura et al, 1992; Thompson et al, 1993).

In past studies using conventional lavage cytology in patients with a complete tumour resection, peritoneal carcinosis has been shown to be more frequent when free peritoneal tumour cells were detected preoperatively (Nakajima et al, 1978; Koga et al, 1984; Asao et al, 1989; Jaehne et al, 1989; Iitsuka et al, 1990; Nishiyama et al, 1995; Bonenkamp et al, 1996). For this reason, it was recently proposed on a trial basis by the International Union Against Cancer (UICC) (UICC, 1993) that tumours with conventional cytological evidence of peritoneal involvement should be classified as pM1, with the provision that this is a cytological finding (Hermanek and Wittekind, 1995).

In general, the sensitivity for the detection of malignant cells in effusions can be increased by a factor of two to three using immunocytology (Johnston et al, 1987; Lidang Jensen and Johansen, 1994). Specifically, very high degrees of sensitivity and specificity can be achieved using the antibody Ber-Ep4 (Latza et al, 1990; De Angelis et al, 1992; Stoop et al, 1992; Diaz-Arias et al, 1993; Robinson and Royston, 1993). This antibody recognizes epithelial glycoproteins 34 and 49 kDa in size, found on the epithelial cell membrane, which correspond to nidogen, a matrix adhesion protein (Simon et al, 1990).

Using Ber-Ep4 for tumour cell detection in peritoneal lavage specimens, we carried out the present prospective study on a large group of patients with completely resected gastric carcinoma to answer the following questions: (1) how frequent is FPTC in completely resected gastric carcinoma?; (2) does FPTC predict the likelihood or pattern of tumour recurrence?; and (3) what impact does FPTC have on patient survival?

*Received 3 April 1998*
*Revised 9 June 1998*
*Accepted 10 July 1998*

*Correspondence to:* H Nekarda, Department of Surgery, Klinikum rechts der Isar, Technical University of Munich, Ismaninger Strasse 22, D-81675 Munich, Germany

**Table 1** Free peritoneal tumour cells (FPTC) compared with TNM category

| Variable | Patients | | FPTC | | | |
|---|---|---|---|---|---|---|
| | *n* | (%) | Negative *n* | Positive *n* | Positive % | *P*-value |
| Tumour (pT) | | | | | | |
| T1 | 24 | (20) | 24 | 0 | – | |
| Mucosa | 10 | (8) | 10 | 0 | – | |
| Submucosa | 14 | (12) | 14 | 0 | – | |
| T2 | 59 | (50) | 48 | 11 | (19) | |
| Muscularis | 25 | (21) | 22 | 3 | (12) | |
| Subserosa | 20 | (17) | 14 | 6 | (30) | |
| Fat tissue | 14 | (12) | 12 | 2 | (14) | |
| T3 | 22 | (19) | 13 | 9 | (41) | |
| T4 | 13 | (11) | 10 | 3 | (23) | 0.006[a] |
| Nodes (pN) | | | | | | |
| N0 | 49 | (41) | 46 | 3 | (6) | |
| N1 | 16 | (14) | 13 | 3 | (19) | |
| N2 | 53 | (45) | 36 | 17 | (32) | 0.004 |
| Metastasis (pM) | | | | | | |
| M0 | 105 | (89) | 86 | 19 | (18) | |
| M1 (lymph) | 13 | (11) | 9 | 4 | (31) | n.s. |

Statistical analysis: chi-squared test; n.s. = not significant ($P > 0.05$); [a]T1/T2/T3/T4; staging of tumour according UICC, 1987 and recommendations of UICC supplement 1993.

**Table 2** Free peritoneal tumour cells (FPTC) compared with stage

| Stage | Category pTNM | Patients | | FPTC | | |
|---|---|---|---|---|---|---|
| | | *n* | (%) | Negative *n* | Positive *n* | Postive (%) |
| IA | pT1 N0 M0 | 23 | (19) | 23 | 0 | – |
| IB | pT1 N2 M0 | – | – | – | – | – |
| | pT2 N0 M0 | 22 | (19) | 20 | 2 | (9) |
| II | pT1 N2 M0 | 1 | (1) | 1 | 0 | – |
| | pT2 N1 M0 | 11 | (10) | 9 | 2 | (18) |
| | pT3 N0 M0 | 4 | (3) | 3 | 1 | (25) |
| IIIA | pT2 N2 M0 | 22 | (19) | 17 | 5 | (23) |
| | pT3 N1 M0 | 4 | (3) | 3 | 1 | (25) |
| | pT4 N0 M0 | – | – | – | – | – |
| IIIB | pT3 N2 M0 | 12 | (10) | 6 | 6 | (50) |
| | pT4 N1 M0 | 1 | (1) | 1 | 0 | – |
| IV | pT4 N2 M0 | 5 | (4) | 3 | 2 | (40) |
| | pT1–4 N1–2 M1 | 13 | (11) | 9 | 4 | (31) |

Statistical analysis: chi-squared test; $P = 0.009$: IA/IB/II/IIIA/IIIB/IV; staging of tumour according to UICC, 1987.

### MATERIALS AND METHODS

### Patient study group

Between June of 1987 and December of 1990, 241 patients were operated upon for a histologically confirmed gastric adenocarcinoma at the Department of Surgery of the Technical University of Munich. These patients are a subgroup of a larger study group which was previously published as part of the German Gastric Carcinoma Study Group (GGCSG) (Roder et al, 1993). One hundred and eighteen patients were included in this prospective study who fulfilled the following criteria: (1) an intraoperative peritoneal lavage yielding a sample which was sufficient for diagnosis; (2) complete macroscopic and histological removal of tumour (R0 resection); (3) no preoperative chemotherapy; (4) a post-operative survival of at least 3 months (to eliminate post-operative complications from survival analysis); and (5) no other primary malignant tumour. These 118 patients included 39 female and 79 male patients with an average age of 59 (range 31–79).

### Operative procedure

The operative procedure was determined by the stage, location and histological Laurèn type according to the standardized protocols of

 © Cancer Research Campaign 1999

the GGCSG (Roder et al, 1993; Siewert et al, 1993). Twenty-one patients (18%) with a T1 or T2 tumour of the intestinal Laurèn type in the distal third of the stomach received a subtotal gastric resection. Fifty-one patients (43%) had a standard total gastrectomy. Forty patients (34%) had an extended gastrectomy. Of these 40 patients, 20 (50%) had a transhiatal, 15 (38%) a left regional and five (12%) both a transhiatal and left regional extension. The remaining six patients (5%) had a resection of their gastric remnant because of a stump carcinoma. Using the recommendations of the Japanese Research Society for Gastric Cancer (JRSGC) (1981), the lymph nodes of compartments I and II were completely removed by an 'en bloc' dissection. The lymph nodes of the hepatoduodenal ligament (station 12) and the retroduodenal lymph nodes (station 13) of compartments III were routinely dissected in patients with advanced (T2–T4) cancers (Siewert et al, 1993).

### Staging and pathological data

The histopathological evaluation of the specimen followed the recommendations of the GGCSG (Roder et al, 1993) with the TNM classification performed according to the guidelines of the UICC (UICC, 1993). Twenty-three of the tumours (19%) were classified as stage IA, 22 (19%) as IB, 16 (14%) as II, 26 (22%) as IIIA, 13 (11%) as IIIB and 18 (15%) as stage IV tumours (see Table 2).

Pathological data included the location, size, histological type according to the WHO (Watananbe et al, 1990), Borrmann (1926) and Laurèn (1965) classifications, grade of the primary tumour and the presence or absence of vessel or perineural invasion, and lymphangiosis carcinomatosa.

### Follow-up data

None of the patients received adjuvant therapy. Approximately 40% of the patients were examined for tumour recurrence in the tumour follow-up programme of the Department of Surgery at the Klinikum rechts der Isar [including a clinical examination, routine and tumour marker laboratory studies, abdominal ultrasound, endoscopy, computerized tomography (CT) and bone scans] in 3-month intervals during the first post-operative year and in 6-month intervals thereafter. Suspected recurrent lesions were confirmed histologically whenever possible. The remaining patients were examined by outside physicians according to this same protocol and the data collected at the Klinikum rechts der Isar. The follow-up data included the location and time of the first tumour recurrence, additional therapy given, survival time, and date and cause of death. None of the patients was lost to follow-up. Fifty-seven patients (48%) died because of tumour recurrence. The median survival time of these patients was 18 months. At the end of the study period, 54 patients (46%) were still alive, the mean follow-up time for these patients was 64 months with a range from 41 to 84 months. Seven patients (6%) died of other causes (three suicide, two cardiovascular disease, one pneumonia, one iatrogenic oesophagus perforation). All of these seven patients were negative for FPTC. For survival analysis, these patients were treated as censored observations. The median survival time for all patients was 69 months and the calculated 5-year survival rate was 52%.

### Lavage evaluation

The lavage was carried out by introducing 500 ml of Ringer's lactate into the upper abdomen immediately after opening the abdominal cavity, followed by collection of five samples of 60 ml

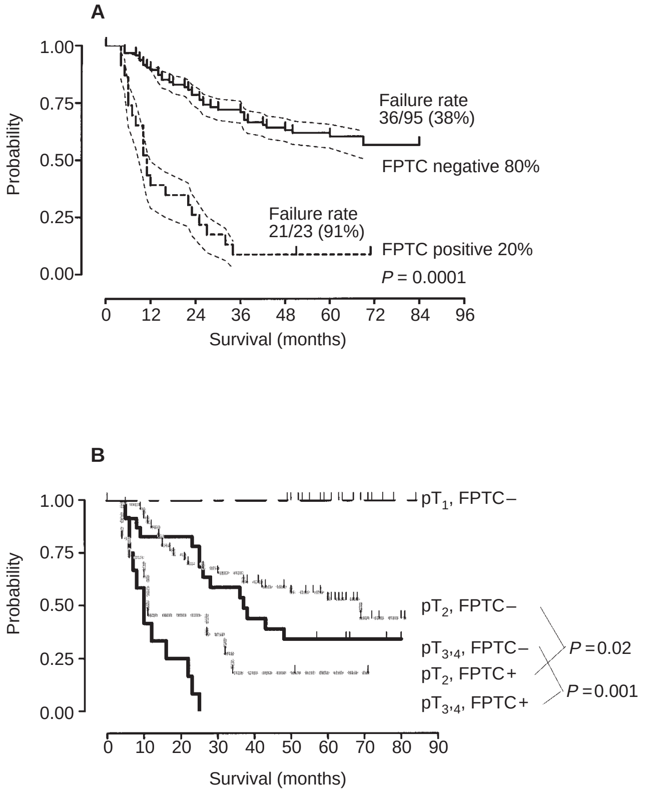

**Figure 1** Kaplan–Meier curves (**A**) FPTC vs. overall survival for the 118 completely resected (R0) gastric carcinoma patients and (**B**) survival curves divided according to pT category. (**A**) Of the 95 patients negative for FPTC, 36 died during the study period, resulting in a predicted 5-year survival probability of 60%. Of the 23 FPTC-positive patients, 21 died during the same period for a predicted 5-year survival probability of 8%. The differences in survival for the FPTC-positive and FPTC-negative groups were statistically significant ($P = 0.0001$). The dotted lines correspond to the 95% confidence intervals. (**B**) None of the pT1 (early gastric carcinoma) cases had FPTC. Within the pT2 category (no histological serosal tumour involvement), the difference in survival between the FPTC-negative and FPTC-positive cases was statistically significant ($P = 0.02$). The same was true for the cases with histological serosal tumour involvement (pT3,4) ($P = 0.001$). For the FPTC-negative subgroup (pT2 FPTC – and pT3,4 FPTC–), survival for the subgroups with or without serosal tumour involvement was not statistically different. This was also true for the FPTC-positive groups, although a tendency ($P = 0.09$) towards a difference in survival was seen comparing cases with or without serosal tumour involvement each from the subphrenic space. For anticoagulation, EDTA at a final concentration of 1 mg $ml^{-1}$ was added.

The samples were centrifuged for 20 min at 1500 r.p.m., haemolysed with 15 ml ammonium chloride buffer (0.8%) and washed with RPMI-1640 solution (ICN Biomedical, Ohio, USA). Nine cytospins (Rotanta P, Hettich, Germany) were prepared with approximately $4 \times 10^4$ cells per slide. The slides were dried overnight at room temperature and fixed for 10 min in acetone solution before staining or storage (–80°C). One slide was stained with a modified rapid Giemsa/May–Gruenwald staining (Diff-Quik; Baxter Diagnostics, Germany), and eight slides with the alkaline phosphatase anti-alkaline phosphatase (APAAP) protocol as previously described (Nakamura et al, 1994) with MCA Ber-Ep4 (Dako, Hamburg, Germany) as the primary antibody in a 1:80 dilution followed by rabbit anti-mouse IgG secondary antibody (Z 259, Dako) in a 1:10 dilution with RPMI and inactivated human serum at 1:8. The APAAP complex was used at a dilution of 1:160

© Cancer Research Campaign 1999

(Dianova, Hamburg, Germany). Meyer's haematoxylin was used as the counterstain. Controls consisted of primary antibody omission [phosphate-buffered saline (PBS) buffer] as the negative control and MOPC-21 (M-9269, Sigma, Munich, Germany) antibody 1:400 as a control for non-specific binding for each set of reactions. Strong membrane and nucleus-associated granular cytoplasmic staining of cells by Ber-Ep4 was considered to be a positive reaction. In addition, to classify a sample as positive for FPTC, the conventional cytological criteria of malignancy were required to be present in a stained cell. On the basis of these criteria, staining patterns were divided into four groups: negative for Ber-Ep4, positive for Ber-Ep4 but lacking cytological criteria of malignancy, isolated cells positive for Ber-Ep4 with cytological criteria of malignancy and numerous cells [> 5 cells per high power field (40×)] positive for Ber-Ep4 with cytological criteria of malignancy.

### Statistics

Comparison of frequency in group comparisons was performed using the chi-squared test. The probability of survival was calculated for the different subgroups by the Kaplan–Meier method (Kaplan and Meier, 1958). Statistical differences in survival were evaluated by the log-rank test. All tests were performed at a significance level of $P < 0.05$. Prognostic factors were evaluated by Cox's proportional hazard model with a log-linear risk function (Cox, 1972). Covariate selection was carried out by a stepwise forward procedure using the SPSS software package (SPSS, Chicago, Illinois, USA).

### RESULTS

### Rate of FPTC

Three of 118 cases (3%) were diagnosed as having FPTC in lavage by routine cytologic methods. Seven additional cases (6%) were diagnosed as suspicious for FPTC and 108 (91%) were diagnosed as negative. All three positive, four of the suspected and 16 of the negative cases had FPTC by immunocytochemistry in the lavage specimens, a total of 23 of the 118 (20%) cases. There were 17 additional cases (14%) in which cells with positivity for Ber-Ep4 were seen, but lacked the necessary cytological criteria of malignancy. These cases were classified as negative for FPTC, and consisted of cases in which staining for Ber-Ep4 was seen as weak cell membrane staining of mesothelial cells or positively stained debris within macrophages. Numerous cells (> 5 cells per high

**Table 3** Free peritoneal tumour cells compared with histopathology

| Variable | Patients | | FPTC | | | |
|---|---|---|---|---|---|---|
| | *n* | (%) | Negative *n* | Positive *n* | Positive (%) | *P*-value |
| WHO classification | | | | | | |
| Tubular | 60 | (51) | 52 | 8 | (13) | |
| Papillary | 7 | (6) | 5 | 2 | (29) | |
| Mucinous | 2 | (2) | 2 | 0 | – | |
| Signet ring cell | 32 | (27) | 24 | 8 | (25) | |
| Undifferentiated | 17 | (14) | 12 | 5 | (29) | n.s. |
| Laurèn classification | | | | | | |
| Intestinal type | 65 | (55) | 57 | 8 | (12) | |
| Nonintestinal type | 53 | (45) | 38 | 15 | (28) | 0.03 |
| Grade | | | | | | |
| G1,2 | 31 | (27) | 30 | 1 | (3) | |
| G3 | 70 | (59) | 52 | 18 | (26) | |
| G4 | 17 | (14) | 13 | 4 | (24) | n.s. |
| Primary tumour lymphatic invasion | | | | | | |
| No | 81 | (69) | 70 | 11 | (14) | |
| Yes | 37 | (31) | 25 | 12 | (32) | 0.02 |
| Perigastric adipose tissue lymphatic invasion | | | | | | |
| No | 103 | (87) | 83 | 20 | (19) | |
| Yes | 15 | (13) | 12 | 3 | (20) | n.s. |
| Perigastric adipose tissue metastasis | | | | | | |
| No | 112 | (95) | 93 | 19 | (17) | |
| Yes | 6 | (5) | 2 | 4 | (67) | 0.003 |
| Perineural invasion | | | | | | |
| No | 110 | (93) | 88 | 22 | (20) | |
| Yes | 8 | (7) | 7 | 1 | (13) | n.s. |
| Angioinvasion | | | | | | |
| No | 114 | (97) | 92 | 22 | (19) | |
| Yes | 4 | (3) | 3 | 1 | (25) | n.s. |

 © Cancer Research Campaign 1999

**Table 4** Multivariate survival analysis (Cox regression model) with grouped variables found to be significant by univariate analysis

| Variable | Category | Univariate analysis *P*-value* | Multivariate analysis | | Confidence interval |
|---|---|---|---|---|---|
| | | | *P*-value* | Relative risk | |
| Tumour (pT)* | T1,2/T3,4 | 0.001 | n.s. | – | – |
| Nodes (pN) | N0/N1,2 | < 0.0001 | 0.001 | 6.63 | 2.30–19.11 |
| Metastasis [pM (lymph)] | M0/M1 | 0.002 | 0.037 | 2.20 | 1.05–4.64 |
| Laurèn classification | Intestinal/ non-intestinal | 0.05 | n.s. | – | – |
| Primary tumour lymphatic invasion | Yes/no | < 0.0001 | n.s. | – | – |
| Perigastric adipose tissue lymphatic invasion | Yes/no | 0.002 | 0.004 | 2.86 | 1.41–5.79 |
| Perigastric adipose tissue metastasis | Yes/no | 0.0001 | n.s. | – | – |
| FPTC | Negative/positive | < 0.0001 | < 0.0001 | 4.48 | 2.34–8.57 |

*n.s, not significant ($P > 0.05$).

power field) were only found in two cases, both of which were also positive by conventional cytology.

### Comparison to TNM category (Table 1)

There were no positive pT1 cases. The proportion of positive cases increased from pT2 (19%) to pT3 (41%) and decreased in the pT4 category (23%). The differences were significant. This may be explained by cases in the pT4 category which had direct tumour extension into neighbouring structures, but no exposed serosal surfaces involved by tumour. The same correlation was also reflected by the rates of FPTC in correlation with local tumour invasion depth in the wall of the stomach from muscularis propria (12%), subserosa (30%) and serosa (41%), whereas only 14% of cases which infiltrated perigastric fatty tissue directly had free tumour cells. The proportion of positive cases also increased significantly according to pN category, with 6% of pN0 to 32% in the pN2 cases. In the pM category, a higher percentage of pM1 (lymph) cases had FPTC, but this did not reach statistical significance because of the small number of cases in this group.

### Comparison to stage (Table 2)

There was a significance for increased incidence of FPTC with advancing tumour stage. Further, within the pT2 or pT3 categories, there was a marked increase in the frequency of FPTC with increasing pN category. For example, in the pT2 category, there was an increased rate of FPTC from 9% in the pN0 to 23% in the pN2 category, and for the pT3 category from 25% to 50% respectively.

### Comparison to histological features (Table 3)

The presence of free metastases in perigastric adipose tissue, which occurred in six cases (5%) seems to be associated with FPTC (four out of six cases, 67%). Lymphatic vessel invasion at the border of the primary tumour (primary tumour lymphatic invasion) was diagnosed in 37 cases (31%) and was also positively associated with FPTC (32% compared with 14%). As for the Laurèn classification, the non-intestinal type tumours had a significantly higher frequency of FPTC than the intestinal type tumours (28% compared with 12%).

No correlation with the frequency of FPTC was seen according to the site of the tumour, WHO histological classification, grade, perineural or vessel invasion in the primary tumour or lymphatic invasion of perigastric adipose tissue. However, the rate of FPTC was significantly higher when more than two regions of the stomach were involved by tumour (41% compared with 16%). In the 47 cases (40%) with tumour size greater than 6 cm, significantly more patients (30%) had FPTC than in the cases with a smaller tumour (13%). Macroscopically diagnosed infiltration of the serosa by the surgeon was also statistically significantly correlated with a higher percentage of FPTC (7 out of 17 cases, 41% compared with 16 of 101 cases, 16%). There was also a higher rate of FPTC (4 out of 11 cases, 36%) of the exophytic type (Borrmann type I) than the scirrhous infiltrating type (Borrmann type IV) (8 out of 20 cases, 40%) and the Borrmann types II and III (11 out of 68 cases, 16%), differences which were not statistically significant however (results not shown).

### Survival analysis

Overall, 21 of the 23 patients with FPTC (91%) and 36 of the 95 patients (38%) without FPTC died of tumour recurrence ($P < 0.0001$) during the observation time. The median survival time for the FPTC-positive patients was 11 months and for FPTC-negative patients more than 72 months, with an estimated 5-year survival rate of 8% and 60%, respectively, as calculated by Kaplan–Meier analysis (Figure 1A).

Figure 1B shows the survival data divided according to pT category. In the pT2 subgroup of 59 patients (without early carcinoma and no histological serosal infiltration or perforation), there was a significant survival advantage for the 48 FPTC-negative patients compared with the 11 FPTC-positive patients. Of the 48 FPTC-negative pT2 patients, 22 (46%) died because of tumour recurrence, with a median survival time of 70 months, compared with 9 out of 11 FPTC-positive pT2 patients (82%), who died with a median survival of 11 months.

For the 35 pT3 and 4 patients (histological serosal tumour involvement), an equally high survival difference was seen between the FPTC-positive and -negative subgroups. The 23 FPTC negative pT3 and 4 patients (66%) had a median survival of 37 months and an estimated 5-year survival probability of 34%.

*© Cancer Research Campaign 1999*

**Table 5** Comparison of FPTC with year of death and location of relapse

| Year of death: | | Year 1 | | Year 2 | | Year 3 | | Year 4 | |
|---|---|---|---|---|---|---|---|---|---|
| **Location** | **Patients** *n* **(%)** | **FPTC –** *n* **= 12** | **FPTC +** *n* **= 14** | **FPTC –** *n* **= 11** | **FPTC +** *n* **= 3** | **FPTC –** *n* **= 7** | **FPTC +** *n* **= 4** | **FPTC –** *n* **= 65** | **FPTC +** *n* **= 2** |
| Relapse | 57 (48) | [24 (42%)] | | [12 (21%)] | | [11 (19%)] | | [10 (17%)] | |
| Peritoneal carcinosis | 18 (32) | 1 | 8 | 3 | 1 | 2 | 1 | 2 | – |
| Lymph node metastasis | 13 (23) | 3 | 2 | 3 | 1 | 2 | – | 2 | – |
| Distant metastasis | 18 (32) | 4 | 4 | 2 | 1 | 1 | 3 | 3 | – |
| Extraluminal relapse | 5 (9) | 1 | – | – | – | 1 | – | 3 | – |
| Intraluminal relapse | 3 (5) | 1 | – | 1 | – | 1 | – | – | – |
| No relapse | 61 (52) | 2[a] | – | 2[a] | – | – | – | 52 + 3[a] | 2 |

[a]Death of other causes.

There were 14 out of 23 (61%) recurrence-related deaths in this group. All 12 of the FPTC-positive pT3 and 4 patients (34%) died of tumour recurrence within the first 2 post-operative years.

After the first post-operative year, there was a tendency for FPTC-positive but serosa-negative patients (pT2) to have a better outcome than FPTC-positive patients with serosal involvement (pT3 and 4). No prognostic difference was seen in the FPTC-negative group with regards to the presence or absence of serosal involvement (Figure 1B).

### Univariate and multivariate Cox analysis (Table 4)

The pT, pN and pM categories, the Laurèn classification, involvement of lymphatics (both in the primary tumour and perigastric adipose tissue), free metastases in perigastric adipose tissue and FPTC were found by univariate analysis to be significantly correlated with survival. The Borrmann and WHO classifications, perineural or vascular invasion, the site of tumour and the surgeon's judgement of serosal tumour invasion were not correlated with survival.

There was a significant survival advantage for the FPTC-negative compared with FPTC-positive patients in the subgroups: pN0, pN+, pM0, stage I/II, stage III/IV, grades 3/4, intestinal Laurèn type, non-intestinal Laurèn type, tumour diameter less than or equal to 6 cm, tumour diameter greater than 6 cm, and the presence or absence of lymphangiosis (results not shown).

By multivariate Cox survival analysis, independent prognostic factors for survival were pN category, FPTC, mesenteric lymphatic vessel invasion, distant lymph node metastases [pM (lymph)] in that order, with relative risk rates of 6.6, 4.5, 2.9 and 2.2 respectively (Table 4).

### FPTC and recurrence pattern

Table 5 shows the clinically diagnosed site of first recurrence and year of death for the 57 patients (48%) who died of recurrent disease during the follow-up period. Two of the 23 FPTC-positive patients (9%) are still alive after 51 and 70 months. These patients were both staged pT2 pN2 pM0 with local wall invasion extending to the subserosa and muscularis propria respectively. Thirty-six patients (38%) of the FPTC-negative patients died of recurrent tumour. For the 4-year follow-up period, the positive predictive value of FPTC was 91% for death because of tumour recurrence, whereas the negative predictive value was 62% for a tumour-free survival. The sensitivity of FPTC for the prediction of tumour recurrence was low at 37%, whereas its specificity of 97% was very high. Comparing the follow-up years, twice as many patients died in the first post-operative year (42%) as in the succeeding 3 years. After the first year, a constant death rate of approximately 20% was seen. Approximately one-third of the deaths were the result of peritoneal carcinosis and one-third the result of distant metastases respectively. Of the remaining one-third, two-thirds of the patients had progression of tumour in the remaining lymph nodes of compartment III or more distant lymph node regions, and only a third were due to local recurrence. Of the patients who died because of peritoneal carcinosis in the first year after surgery, there was a significantly ($P = 0.02$) higher percentage of FPTC-positive patients (8 out of 14, 57%) than FPTC-negative patients (1 out of 12, 8%). Within the subgroups pT2 and pT3 and 4, the same statistical relation was seen. However, only 10 out of 18 patients (55%) who died because of peritoneal carcinosis had FPTC in their lavage specimens. After the first year of follow-up, there was no longer a correlation between the site of recurrence and the finding of FPTC.

### DISCUSSION

### FPTC: frequency, conventional cytology

The detection of isolated tumour cells for the diagnosis of malignant ascites is a routine procedure in the clinical practice of oncology and is a finding which is equivalent to the diagnosis of disseminated peritoneal carcinosis. Prognostically, such a peritoneal carcinosis represents an incurable situation for gastrointestinal carcinoma (Spratt, 1986; Parsons et al, 1996). Approximately 40–50% of patients who have a resection for gastric carcinoma have local infiltration of the gastric serosa (Nakamura et al, 1992; Roder et al, 1993), and one might expect that cells from the gastric serosa would desquamate into the peritoneal space and be cytologically detectable in most of these cases

 *© Cancer Research Campaign 1999*

(Kiyasu et al, 1981). However, it has been demonstrated in cases of definite tumour involvement of the gastric serosa without metastatic peritoneal implants, that only from 12% (Bonenkamp et al, 1996) to 22% (Chen and Liu, 1994) of cases have detectable free tumour cells. With an established peritoneal carcinosis, the rate of positive cytological diagnoses ranges from 50% (Jaehne et al, 1989) to 81% (Kaibara et al, 1987), with the provision that the rate of positive diagnosis is dependent on the extent of peritoneal carcinosis and, more importantly, on the presence of ascites.

In our study, we found a rate of FPTC in conventional cytology of 3% with an additional 6% of cases which were diagnosed as suspicious for malignancy. In a subgroup of the Dutch Gastric Cancer Study (Bonenkamp et al, 1996), which is presently the single largest study of this topic in the Western world, among completely (R0-UICC) resected patients, there was detection rate of FPTC in 4% of patients, with an additional 1% diagnosed as suspicious for malignancy. In a smaller series, in tumours operated upon according to the guidelines of the GGCS (Roder et al, 1995), as was our study, Jaehne et al (1989) found a rate of 11% for FPTC. In the remaining studies of FPTC in gastric carcinoma, which are almost exclusively from Asia, rates of 14% (Koga et al, 1984) and 22% (Chen and Liu, 1994) are quoted for patients who have had a ‘curative’ resection according to the guidelines of the JRSGC (Japanese Research Society for Gastric Cancer, 1981). In patient collectives with ‘non-curative’ resections (R1, 2-UICC), rates of FPTC ranging from 23% (Bonenkamp et al, 1996) to 59% (Ikeguchi et al, 1994) have been found.

In contrast, FPTC can be found in cases with a normal-appearing gastric serosa and a complete gastric resection. In the series of Bonenkamp (1996), in which there were 217 patients, a rate of 1% FPTC was found in such cases. In clinically suspicious, but histologically non-infiltrated serosa (S1 according to the JRSGC), rates for comparable patient groups of 3% (Koga et al, 1984; Boku et al, 1990) have been reported, whereas others found no positive cases (Chen and Liu, 1994).

Two pathogenetic mechanisms can be hypothesized to explain the finding of FPTC even when the local gastric serosa is not histologically involved by tumour. First, small numbers of individual tumour cells, not detectable by routine histological methods, could be desquamated into the peritoneum from the serosa adjacent to the tumour (Kiyasu et al, 1981). Alternatively, spread of tumour cells in the mesenteric lymphatics with seeding into the peritoneal space through so-called ‘gaps’ in the mesenteric lymphatics in association with milky spots has been hypothesized (Spratt, 1986; Hagiwara et al, 1993). In contrast, as mentioned above, not every case in which the serosa is involved by tumour shows FPTC. It is probable that not every tumour cell on the serosal surface can detach itself, and, even once it is shed, have the ability to survive in the peritoneal space.

### FPTC: immunocytology

In our study, we achieved a tripling of the detection rate of positive cases to 20% in comparison with conventional cytology. This is in line with previous studies which generally have reported a factor of two- to threefold over conventional cytology for tumour cell detection in effusions (Johnston et al, 1987; Lidang Jensen and Johansen, 1994). Among the various antibodies which have been used, Ber-Ep4 (Latza et al, 1990) has shown itself to be highly reliable for the detection of epithelial tumour cells, with a sensitivity of 83–100%, a positive predictive value of 96%, and a false-positive rate of 2–7% (De Angelis et al, 1992; Stoop et al, 1992; Diaz-Arias et al, 1993; Robinson and Royston, 1993; Lidang Jensen and Johansen, 1994).

Juhl et al (1994) and Broll et al (1996) were among the first to report results with immunocytology in peritoneal lavage specimens for a variety of gastrointestinal carcinomas. They used a panel of multiple antibodies [carcinoembryonic antigens (CEA), cytokeratins, mucin antibodies, etc.] and found rates of FPTC of 43% and 69%, respectively, including up to 25% positivity in early carcinomas. In these studies, however, positivity was defined solely as positive staining of cells regardless of cytological features, and correlation with prognosis was not reported.

Our FPTC-positive cases included 57% of the cases which had been classified as suspicious and 13.5% of the cases classified as negative by conventional cytology. These patients showed the same poor prognosis as those which had been classified as positive by conventional cytology. In our study, we also required that the positively stained cells had cytological features of malignancy to avoid false-positive results. In 14% of our cases, we observed clusters of obvious mesothelial cells with a positive, strongly membrane-based stain. Because most of these cases had invasion of the primary tumour to the subserosa, this phenomenon could be due to the epithelial glycoprotein antigen expressed by tumour cells which was bound by membrane proteins of the mesothelial cells in the process of adhesion. Epithelial glycoprotein (Egp-34) has not been reported to be expressed by mesothelial cells themselves (Latza et al, 1990). Another, more frequent, finding was Ber-Ep4 staining in macrophages, which would also have been a source of false-positive diagnoses if cytological criteria for malignancy had not been used to identify the tumour cells.

### Correlation of FPTC with histopathological features

All previous studies of FPTC in gastric carcinoma have shown a strong correlation between the extent of local tumour infiltration of the gastric serosa and the FPTC rate. When tumour infiltration of the gastric serosa was measured planimetrically (Kaibara et al, 1987), a significant difference was found between 10–15 $cm^2$ and 20 $cm^2$. In the subgroup T3 N0–2 M0, Abe et al (1995) found a prognostically relevant cut-off point of 3 cm diameter (histologically determined) tumour serosal involvement, for which the rate of FPTC rose from 7% to 32%. Bonenkamp et al (1996) could show that the risk for the development of FPTC was most strongly dependent on serosal involvement by a factor of 11 in comparison with lymph node status or stage. We found a positive correlation between tumour diameter ($< 6$ cm vs. $> 6$ cm) and the rate of FPTC.

As in our study, no previous study using conventional cytology has found FPTC in early gastric carcinoma. Juhl et al (1994) and Broll et al (1996) found rates of FPTC of up to 25% in their cases of early gastric carcinoma, which might be explained by a high false-positive rate.

The positive correlation between FPTC and lymph node status, which we found, has been previously reported by several authors (Chen and Liu, 1994; Ikeguchi et al, 1994; Juhl et al, 1994; Abe et al, 1995; Bonenkamp et al, 1996).

Our study showed a correlation between undifferentiated, non-intestinal or infiltrative classified gastric carcinoma and a higher rate of FPTC. Previous studies have shown a similar correlation between FPTC and the histological classification of the carcinoma

© Cancer Research Campaign 1999

according to Laurèn (Jaehne et al, 1989), Ming (Chen and Liu, 1994; Ikeguchi et al, 1994) or the WHO classification as modified by Sugano (Boku et al, 1990; Iitsuka et al, 1990; Chen and Liu, 1994; Ikeguchi et al, 1994). As in our study, higher rates of FPTC have also been found in the literature for macroscopic Borrmann type IV tumours (Chen and Liu, 1994), but no correlation between FPTC and grade, perineural invasion or vascular invasion has been described in the literature. The positive correlation between FPTC and lymphangiosis in the primary tumour or presence of free metastases in the perigastric adipose tissue has not been previously described.

### Impact of FPTC on survival

We found 5-year survival rates for FPTC-positive compared with FPTC-negative groups of 8% and 60%, similar to most previous studies in which the survival rates for FPTC-positive patients with 'curative' or R0-UICC resections have been found to range between 13% (Koga et al, 1984) and 25% (Boku et al, 1990; Chen and Liu, 1994) compared with FPTC-negative patients who had 5-year survival rates of between 46% (Chen and Liu, 1994) and 54% (Boku et al, 1990). Of the five studies in which a univariate survival analysis was carried out to determine the impact of FPTC, only Abe et al (1995) found no significant difference in survival. No difference in prognosis has been seen in comparison with the numbers of free tumour cells or whether or not the tumour cells were found in clusters or as single cells (Iitsuka et al, 1990).

In the analysis of our subgroups with or without a histologically confirmed involvement of the serosa, we found FPTC in 19% of those with an unremarkable serosa. In terms of survival, FPTC proved to be a more important factor than serosal involvement because, within the FPTC-positive and FPTC-negative subgroups, the presence or absence of serosal involvement had no further impact on prognosis when cases of early gastric carcinoma were excluded. In contrast, the prognosis of all FPTC-positive patients corresponded to the prognosis of patients in our study with pM1-(lymph), who had a median survival time of 27 months.

By multivariate analysis, detection of FPTC was the second strongest prognostic factor after lymph node status. A comparable multivariate analysis has not yet been reported in the literature. In contrast to the results of Abe et al (1995), FPTC was also a highly significant prognostic factor for T3 N0–2 M0 patients in the analysis of our subgroups.

### FPTC and pattern of tumour recurrence

In our study, 57% of the patients who died within the first post-operative year as a result of peritoneal carcinosis had had FPTC. This pattern was significantly different from FPTC-negative cases. However, after the first year, there was no difference in the pattern of tumour recurrence between FPTC-positive and -negative cases. Other studies have also reported that gastric carcinoma patients with free tumour cells in their lavage specimens have, after curative resection, a high likelihood of developing a peritoneal carcinosis (Koga et al, 1984). Again, this is in contrast to the study of Abe et al (1995), who found no correlation between FPTC and recurrence in the form of peritoneal carcinosis (30% in his study).

The FPTC-positive patients in our study who died within the first year were not characterized by particular histopathological or staging parameters, so that they cannot have been identified with any conventional prognostic factors.

### CONCLUSION

Using immunocytology, we found FPTC to have a strong negative, independent impact in patients who had a complete resection of their gastric carcinoma, with an impact similar to the presence of distant nodal metastases (pM1 lymph). A particularly important finding of our study in this regard lies in the detection of FPTC and its attendant poor prognosis in 19% of patients without histological serosal involvement by tumour. Although some authors have recommended the planimetric measurement of tumour involvement of the serosa, our results show that the finding of FPTC has an even greater impact. Boku et al (1990) demonstrated that the prognosis of FPTC-positive patients was similar to that of patients who had a metastatic peritoneal carcinosis (P1,2-JRSGC). This indicates that, in some FPTC-positive cases, a so-called 'occult peritoneal carcinosis (Kiyasu et al, 1981) is present, perhaps represented in our study by the subgroup of FPTC-positive patients who died in the first post-operative year and had a very high risk for the development of peritoneal carcinosis. In other cases, FPTC seems to carry a risk of distant metastatic disease similar to the impact of isolated tumour cells in the bone marrow (Jauch et al, 1996).

On the basis of our findings, we believe that the detection of FPTC might form an important component of preoperative staging, especially when the option of neoadjuvant therapy is to be considered. Our results strongly support the recommendation of the UICC that cases with FPTC should be designated as 'pM1' or, in cases of an otherwise complete resection, that the designation 'R1c' be used because this has a strong impact on the prognosis of the patient.

#### REFERENCES

Abe S, Yoshimura H, Tabara H, Tachibana M, Monden N, Nakamura T and Nagaoka S (1995) Curative resection of gastric cancer: limitation of peritoneal lavage cytology in predicting the outcome. *J Surg Oncol* **59**: 226–229

Allum WH, Powell DJ, McConkey CC and Fielding JW (1989) Gastric cancer: a 25-year review. *Br J Surg* **76**: 535–540

Asao T, Fukuda T, Yazawa S and Nagamachi Y (1989) CEA levels in peritoneal washings from gastric cancer patients as a prognostic guide. *Cancer Lett* **47**: 79–81

Boku T, Nakane Y, Minoura T, Takada H, Yamamura M, Hioki K and Yamamoto M (1990) Prognostic significance of serosal invasion and free intraperitoneal cancer cells in gastric cancer. *Br J Surg* **77**: 436–439

Bollschweiler E, Boettcher K, Hoelscher AH, Sasako M, Kinoshita T, Maruyama K and Siewert JR (1993) Is the prognosis for Japanese and German patients with gastric cancer really different? *Cancer* **71**: 2918–2925

Bonenkamp JJ, van de Velde CJ, Kampschoer GH, Hermans J, Hermanek P, Bemelmans M, Gouma DJ, Sasako M and Maruyama K (1993) Comparison of factors influencing the prognosis of Japanese, German, and Dutch gastric cancer patients. *World J Surg* **17**: 410–414

Bonenkamp JJ, Songun I, Hermans J, Sasako M, Welvaart K, Plukker JT, van Elk P, Obertop H, Gouma DJ and Taat CW (1995) Randomised comparison of morbidity after D1 and D2 dissection for gastric cancer in 996 Dutch patients. *Lancet* **345**: 745–748

Bonenkamp JJ, Songun I, Hermans J and van de Velde CJ (1996) Prognostic value of positive cytology findings from abdominal washings in patients with gastric cancer. *Br J Surg* **83**: 672–674

Borrmann R (1926) Geschwulste des Magens und Duodenums. *Handbuch der Speziellen Pathologischen Anatomie und Histologie*. Henke F and Lubarsch O (eds.), pp. 865. Springer: Berlin

Broll R, Lembcke K, Stock C, Zingler M, Duchrow M, Schimmelpenning H, Strik M, Muller G, Kujath P and Bruch HP (1996) Tumorzelldissemination in das knochenmark und in die peritonealhoehle. Eine immunzytochemische untersuchung an patienten mit einem magen; oder kolerektalen karzinom. *Langenbecks Arch Chir* **381**: 51–58

Chen J and Liu Q (1994) Identification and classification of serosal invasion, as it relates to cancer cell shedding and surgical treatment in gastric cancer. *Semin Surg Oncol* **10**: 107–110

*© Cancer Research Campaign 1999*

Cox D (1972) Regression models and life-tables. *J R Stat Soc* **34**: 187–220
De Angelis M, Buley ID, Heryet A and Gray W (1992) Immunocytochemical staining of serous effusions with the monoclonal antibody Ber-EP4. *Cytopathology* **3**: 111–117
Diaz-Arias AA, Loy TS, Bickel JT and Chapman RK (1993) Utility of BER-EP4 in the diagnosis of adenocarcinoma in effusions: an immunocytochemical study of 232 cases. *Diagn Cytopathol* **9**: 516–521
Hagiwara A, Takahashi T, Sawai K, Taniguchi H, Shimotsuma M, Okano S, Sakakura C, Tsujimoto H, Osaki K and Sasaki S (1993) Milky spots as the implantation site for malignant cells in peritoneal dissemination in mice. *Cancer Res* **53**: 687–692
Hermanek P and Wittekind C (1995) News of TNM and its use for classification of gastric cancer. *World J Surg* **19**: 491–495
Iitsuka Y, Shiota S, Matsui T, Murata Y, Kimura A and Koga S (1990) Relationship between the cytologic characteristics of intraperitoneal free cancer cells and the prognosis in patients with gastric cancer. *Acta Cytol* **34**: 437–442
Ikeguchi M, Oka A, Tsujitani S, Maeta M and Kaibara N (1994) Relationship between area of serosal invasion and intraperitoneal free cancer cells in patients with gastric cancer. *Anticancer Res* **14**: 2131–2134
Jaehne J, Meyer HJ, Soudah B, Maschek H and Pichimayr R (1989) Peritoneal lavage in gastric carcinoma. *Dig Surg* **6**: 26–28
Japanese Research Society for Gastric Cancer (1981) The general rules for the gastric cancer study in surgery and pathology. II. Histological classification of gastric cancer. *Jpn J Surg* **11**: 140–145
Jauch KW, Heiss MM, Gruetzner U, Funke I, Pantel K, Babic R, Eissner HJ, Riethmueller G and Schildberg FW (1996) Prognostic significance of bone marrow micrometastases in patients with gastric cancer. *J Clin Oncol* **14**: 1810–1817
Johnston WW, Szpak CA, Thor A, Simpson JF and Schlom J (1987) Applications of immunocytochemistry to clinical cytology. *Cancer Invest* **5**: 593–611
Juhl H, Stritzel M, Wroblewski A, Henne-Bruns D, Kremer B, Schmiegel W, Neumaier M, Wagener C, Schreiber HW and Kalthoff H (1994) Immunocytological detection of micrometastatic cells: comparative evaluation of findings in the peritoneal cavity and the bone marrow of gastric, colorectal and pancreatic cancer patients. *Int J Cancer* **57**: 330–335
Kaibara N, Iitsuka Y, Kimura A, Kobayashi Y, Hirooka Y, Nishidoi H and Koga S (1987) Relationship between area of serosal invasion and prognosis in patients with gastric carcinoma. *Cancer* **60**: 136–139
Kaplan EL and Meier P (1958) Nonparametric estimation from incomplete observations. *J Am Stat Assoc* **53**: 457–481
Kiyasu Y, Kaneshima S and Koga S (1981) Morphogenesis of peritoneal metastasis in human gastric cancer. *Cancer Res* **41**: 1236–1239
Koga S, Kaibara N, Iitsuka Y, Kudo H, Kimura A and Hiraoka H (1984) Prognostic significance of intraperitoneal free cancer cells in gastric cancer patients. *J Cancer Res Clin Oncol* **108**: 236–238
Latza U, Niedobitek G, Schwarting R, Nekarda H and Stein H (1990) Ber-EP4: new monoclonal antibody which distinguishes epithelia from mesothelia. *J Clin Pathol* **43**: 213–219
Lauren P (1965) The two histological main types of gastric carcinoma: diffuse and so-called intestinal-type carcinoma. *Acta Pathol Microbiol Scand* **64**: 31–49
Lidang Jensen M and Johansen P (1994) Immunocytochemical staining of serous effusions: an additional method in the routine cytology practice? *Cytopathology* **5**: 93–103
Nakajima T, Harashima S, Hirata M and Kajitani T (1978) Prognostic and therapeutic values of peritoneal cytology in gastric cancer. *Acta Cytol* **22**: 225–229
Nakamura K, Ueyama T, Yao T, Xuan ZX, Ambe K, Adachi Y, Yakeishi Y, Matsukuma A and Enjoji M (1992) Pathology and prognosis of gastric carcinoma. Findings in 10 000 patients who underwent primary gastrectomy. *Cancer* **70**: 1030–1037
Nakamura T, Nekarda H, Hoelscher AH, Bollschweiler E, Harbeck N, Becker K, Siewert JR and Harbec NH (1994) Prognostic value of DNA ploidy and c-erbB-2 oncoprotein overexpression in adenocarcinoma of Barrett's esophagus. *Cancer* **73**: 1785–1794
Nishiyama M, Takashima I, Tanaka T, Yoshida K, Toge T, Nagata N, Iwamori S and Tamura Y (1995) Carcinoembryonic antigen levels in the peritoneal cavity: useful guide to peritoneal recurrence and prognosis for gastric cancer. *World J Surg* **19**: 133–137
Noguchi Y, Imada T, Matsumoto A, Coit DG and Brennan MF (1989) Radical surgery for gastric cancer. A review of the Japanese experience. *Cancer* **64**: 2053–2062
Parsons SL, Watson SA and Steele RJ (1996) Malignant ascites. *Br J Surg* **83**: 6–14
Robinson RJ and Royston D (1993) Comparison of monoclonal antibodies AUA1 and BER EP4 with anti-CEA for detecting carcinoma cells in serous effusions and distinguishing them from mesothelial cells. *Cytopathology* **4**: 267–271
Roder JD, Bottcher K, Siewert JR, Busch R, Hermanek P and Meyer HJ (1993) Prognostic factors in gastric carcinoma. Results of the German Gastric Carcinoma Study 1992. *Cancer* **72**: 2089–2097
Roder JD, Bonenkamp JJ, Craven J, van de Velde CJ, Sasako M, Bottcher K and Stein HJ (1995) Lymphadenectomy for gastric cancer in clinical trials: update. *World J Surg* **19**: 546–553
Siewert JR, Bottcher K, Roder JD, Busch R, Hermanek P and Meyer HJ (1993) Prognostic relevance of systematic lymph node dissection in gastric carcinoma. German Gastric Carcinoma Study Group. *Br J Surg* **80**: 1015–1018
Siewert JR, Kestlmeier R, Busch R, Bottcher K, Roder JD, Muller J, Fellbaum C and Hofler H (1996) Benefits of D2 lymph node dissection for patients with gastric cancer and pN0 and pN1 lymph node metastases. *Br J Surg* **83**: 1144–1147
Simon B, Podolsky DK, Moldenhauer G, Isselbacher KJ, Gattoni-Celli S and Brand SJ (1990) Epithelial glycoprotein is a member of a family of epithelial cell surface antigens homologous to nidogen, a matrix adhesion protein. *Proc Natl Acad Sci USA* **87**: 2755–2759
Spratt JS (1986) Peritoneal carcinomatosis: anatomy, physiology, diagnosis, management. *Curr Probl Cancer* **10**: 558–584
Stoop JA, Hendriks JG and Berends D (1992) Identification of malignant cells in serous effusions using a panel of monoclonal antibodies Ber-EP4, MCA-b-12 and EMA. *Cytopathology* **3**: 297–302
Thompson GB, van Heerden JA and Sarr MG (1993) Adenocarcinoma of the stomach: are we making progress? *Lancet* **342**: 713–718
UICC (1993) *TNM Atlas*: *an Illustrated Guide to the TNM/pTNM Classification of Malignant Tumors*, pp. 359. Springer-Verlag: Berlin
Watananbe H, Jass JR, and Sobin LH (1990) *WHO Histological Typing of Oesophageal and Gastric Tumors*. Springer: Berlin

© Cancer Research Campaign 1999
